# Suppression of *Laccase 2* severely impairs cuticle tanning and pathogen resistance during the pupal metamorphosis of *Anopheles sinensis* (Diptera: Culicidae)

**DOI:** 10.1186/s13071-017-2118-4

**Published:** 2017-04-04

**Authors:** Ming-Hui Du, Zheng-Wen Yan, You-Jin Hao, Zhen-Tian Yan, Feng-Ling Si, Bin Chen, Liang Qiao

**Affiliations:** Chongqing Key Laboratory of Vector Insects; Institute of Entomology and Molecular Biology; College of Life Sciences, Chongqing Normal University, Chongqing, 401331 China

**Keywords:** *Anopheles sinensis*, *Laccase 2*, Phenol oxidases, Cuticle tanning, Melanization, Immunity, Pupal metamorphosis

## Abstract

**Background:**

Phenol oxidases (POs) catalyze the oxidation of dopa and dopamine to melanin, which is crucial for cuticle formation and innate immune maintenance in insects. Although, Laccase 2, a member of the PO family, has been reported to be a requirement for melanin-mediated cuticle tanning in the development stages of some insects, whether it participates in cuticle construction and other physiological processes during the metamorphosis of mosquito pupae is unclear.

**Methods:**

The association between the phenotype and the expression profile of *Anopheles sinensis Laccase 2* (*AsLac2*) was assessed from pupation to adult eclosion. Individuals showing an expression deficiency of *AsLac2* that was produced by RNAi and their phenotypic defects and physiological characterizations were compared in detail with the controls.

**Results:**

During the dominant expression period, knockdown of *AsLac2* in pupae caused the cuticle to be unpigmented, and produced thin and very soft cuticles, which further impeded the eclosion rate of adults as well as their fitness. Moreover, melanization immune responses in the pupae were sharply decreased, leading to poor resistance to microorganism infection. Both the high conservation among *Laccase 2* homologs and a very similar genomic synteny of the neighborhood in *Anopheles* genus implies a conservative function in the pupal stage.

**Conclusions:**

To our knowledge, this is the first study to report the serious phenotypic defects in mosquito pupae caused by the dysfunction of *Laccase 2*. Our findings strongly suggest that *Laccase 2* is crucial for *Anopheles* cuticle construction and melanization immune responses to pathogen infections during pupal metamorphosis. This irreplaceability provides valuable information on the application of *Lacccase 2* and/or other key genes in the melanin metabolism pathway for developing mosquito control strategies.

**Electronic supplementary material:**

The online version of this article (doi:10.1186/s13071-017-2118-4) contains supplementary material, which is available to authorized users.

## Background

Melanin is not only the substrate used for cuticle tanning in insects, but it is also involved in innate immune responses against exogenous pathogen infections through melanotic encapsulation [1–8]. Thus, melanin metabolism determines whether these two essential physiological processes can be normally activated and affect the typical development of insects. For the Mosquitoes, the adults pose a threat to human health because they transmit malignant diseases by biting humans [9–11]. During the pre-developmental stage of the adult, the development status of the mosquito pupae directly affects the eclosion rate, and further affects the population growth rate of adults.

During pupal development, the cuticle gradually darkens and sclerotizes, which provides enough support for pupae to break out of the puparium, protects them from mechanical injuries, and facilitates the emergence of adults; in addition, some intermediates of melanin metabolism are known to be involved in encapsulation, which is helpful for the survival of the pupae in natural ecosystems [7, 11–16]. Therefore, melanin metabolism during the pupal stage is crucial for mosquito development, survival, and reproduction.

The metabolism of melanin with the hydroxylation of tyrosine to dihydroxy phenylalanine (dopa) by the rate-limiting enzyme tyrosine hydroxylase (TH), followed by the decarboxylation of dopa to dopamine by the dopa decarboxylase (DDC) [5, 17–19]. These melanin precursors must be further oxidized by phenol oxidases (POs) to quinones and quinone methides [19–25]. Subsequently, the quinones and quinone methides conjugate with cuticular proteins to construct and tan the cuticle, and are also involved in melanotic encapsulation [3, 5, 14, 15, 17, 18, 22–25]. Laccase, a member of the phenol oxidase family, conservatively contains three cupredoxin-like domains and four copper ions that reside in a T1 copper site and a T2/T3 tricopper center [5, 26]. Laccases oxidize a broad range of substrates, including polyphenol, methoxy-substituted phenol, aminophenol, and phenylenediamine [5, 27–29]. There are two major types of Laccase genes, *Laccase 1* and *Laccase 2*, that have been identified in many insects [30–44]. Laccase 1 has been reported to likely be involved in cuticular sclerotization [33, 35, 37], while Laccase 2 is mainly expressed in the cuticle or egg shell and is directly involved in melanin-mediated cuticle tanning [38–44]. In the red flour beetle, stinkbug, and honey bee, the pupal cuticle or newly-molted adult becomes white and more flexible due to the depletion of Laccase 2 [38, 40, 42]. Additionally, the dysfunction of Laccase 2 in mosquito eggs results in pale and fragile eggshells, which further causes the eventual collapse of the eggs [39]. This evidence suggests that Laccase 2 is an important regulator in melanin synthesis and deposition. Its dysfunction results in melanin precursors that fail to be oxidized, and impairs the pigmentation and sclerotization of the cuticle or exoskeleton. Although the expression of *Laccase 2* is enriched in the epidermis, we speculated that it is also expressed in immune tissues, such as hemolymph or fat body, and may be involved in melanin synthesis, which participates in melanotic encapsulation immune responses. However, knowledge of *Laccase 2* functions in cuticle tanning and pathogen resistance during mosquito pupal development is still limited.

Knockdown of the *Laccase 2* gene (*AsLac2*) in the pupal stage was performed by RNAi to better understand its function in the pupal development of *Anopheles sinensis*. Our results revealed that the pupae with an unpigmented cuticle developed abnormally, had a severe mortality rate, and were susceptible to exogenous microorganism infection. These findings support that *AsLac2* is not only required for cuticle tanning, but also for melanotic immune responses of the malaria vector mosquitoes, *Anopheles sinensis.* Moreover, the high genomic synteny of the neighborhood of *Laccase 2* in species of the genus *Anopheles* may be a strong indicator of the gene functional conservation in the specific developmental stages. The present study deepens our understanding of the function of the *AsLac2* gene in mosquitoes and provides a new reference for mosquito control.

## Methods

### Insect rearing

The *Anopheles sinensis* LS-WX strain was reared at 27 °C with 80% humidity under a 12 h/12 h (light/dark) photoperiod. The larvae in the different developmental stages were fed fry food in clean water, and the adults were provided 10% glucose solution.

### Identification and cloning of *Laccase 2*

BLAST analysis was performed to search homologous Laccase from the *Anopheles sinensis* genome and transcriptome databases using three insect Laccase 2 proteins (Protein ID: AGAP006176-PB, NP_001034487, and BAG70891) as queries. Fragments from the transcriptome data [45] were assembled using the SeqMan program (https://www.dnastar.com/). The signal peptide was predicted by SignalP 4.1 (http://www.cbs.dtu.dk/services/SignalP) and conserved domains were analyzed by SMART (http://smart.embl-heidelberg.de/). Total RNA was extracted from the pupae (after pupation 32 h) using the TRizol reagent (Invitrogen, Shanghai, China) according to the manufacturer’s instructions. Total RNA (1 μg) was reverse-transcribed with random primer using the First-Strand cDNA Synthesis Kit (Takara, Dalian, China). Five pairs of primers were designed based on the putative *AsLac2* sequence to obtain the full open reading frame (ORF). The sequence of 3′UTR was obtained by the rapid amplification of cDNA ends (RACE) technique using the GeneRacer kit (Invitrogen, Shanghai, China). PCR products were isolated and subcloned into PMD-19 vector (Takara, Dalian, China) for sequencing. The primers are listed in Additional file 1: Table S1.

### Phylogenentic and genome synteny analysis

Homologous Laccase were searched in the NCBI database (http://www.ncbi.nlm.nih.gov/) and Vectorbase (http://www.vectorbase.org/) using the BLASTP program. Amino acid sequences of the divergence part in the C-terminus were aligned with MUSCLE (http://www.ebi.ac.uk/Tools/msa/muscle/). The best-fit evolutionary model (WAG + G) and the genetic distance were estimated by MEGA 5.0 (http://www.megasoftware.net/) [46]. Maximum likelihood phylogenetic analysis was conducted by MEGA 5.0 and bootstrap values were obtained based on 1,000 bootstrap replications. Additionally, the *An. gambiae Laccase 2* gene and its adjacent genes were used as the templates to search for Laccase 2 homologs in other insect genomes, and their genome locations and distributions were compared in detail. We selected 7.5 kb upstream DNA sequences of the *Laccase 2* gene to predict *cis*-acting regulatory elements would respond to hormonal signals during pupal development using JASPAR (http://jaspar.genereg.net/) with 90% confidence settings.

### Temporal-spatial expression analysis of *AsLac2*

Three individuals at each sampling point were collected at different development stages (pupae were collected at 0, 8, 16, 24, 32 and 40 h after pupation and the adults were collected at 0, 3, 6, 9, 12, 24, 48 and 72 h after the eclosion) for phenotype observations (Olympus, Shanghai, China) and gene expression analysis of *AsLac2*. The cuticle, fat body, and hemolymph of the pupae were dissected at 32 h after pupation for tissue expression pattern analysis. Gene expression was determined by qRT-PCR as previously described [12]. Each sample was used to perform three biological repeats. The *Ribosomal protein L49* (*RPL49*) gene was used as the internal control (see primers listed in Additional file 1: Table S1).

### Gene function analysis though RNAi-mediated silencing

Double-stranded RNA (dsRNA) of *AsLac2* (ds*Lac2* and ds*Lac2-2*, designed to avoid the off-target effect) were synthesized with a T7 RiboMAX™ Express RNAi System (Promega, Chongqing, China) according to the manufacturer’s instructions. For microinjection, the dsRNA was dissolved in RNase-free water and the concentration was estimated. Based on *AsLac2* temporal expression patterns, each of the pupae was injected with 800 ng of dsRNA (volume: 180–200 nl) into the thorax through the back of the dorsal plate within 2 h after the pupation according to the temporal expression pattern of *AsLac2*. A red fluorescent protein gene (ds*Red*) was used as the control. The tanning degree of the pupae was checked at 38 h after ds*Lac2* or ds*Red* injection, while the checking time for adults was 3 h after the eclosion. Three pupae or adults in the ds*Lac2-* and ds*Red-*injected groups at each sampling point were also collected for qRT-PCR analyses [12]. Each sample was used to perform three biological repeats. The *Ribosomal protein L49* (*RPL49*) gene was used as the internal control. The primers used for dsRNA synthesis are listed in Additional file 1: Table S1.

### Cuticle structure observation by frozen section

The dorsal plates of the adults from ds*Lac2*- and ds*Red-*injected groups were dissected under the microscope (Olympus, Japan). All of the tissue treatments and slice preparations were carried out as previously described [12]. The thickness of each cuticle layer was measured with Image J software (https://imagej.net/Welcome) as previously described [12]. Four equal spaced measurements for each dorsal plate section, and the average value was calculated as the mean thickness in one biological repeat. Four biological replicates were performed in the ds*Lac2*- and the ds*Red*-injected groups, respectively.

### Pathogen infection and in vivo melanization assays

The pathogens, *Serratia marcescens* (*Sm*) and *Bacillus bombyseptie* (*Bb*), were incubated to the logarithmic phase (OD_600_ = 0.6–0.8) in LB medium at 37 °C. Individuals in the ds*Lac2-* (failed to pigment) and the ds*Red-*injected groups were sampled according to the melaninization degree, and then injected with 0.12 μl of bacteria solution at 26 h after pupation, respectively (the colour of the pupa cuticle in the *dsRed* group started to darken, while the cuticle of *Lac2*-silenced pupae failed to tan). The pupal survival rate was recorded every 2 h after being infected. Six pupae at the same developmental stage of the same size were homogenized at 4 °C with 400 μl of PBS (pH = 7.0) and centrifuged (500× *g* for 5 min at 4 °C). Total protein concentrations (8 mg/ml) were determined using the Bradford method (BBI, Hong Kong, China). Melanization reactions were incubated at 30 °C for 3 h, followed by adding 1 mM of phenylthiourea (PTU) to terminate the reactions and A490 nm values were measured to estimate the amount of melanin. Each sample was used to perform three biological repeats.

## Results

### Characteristics of *AsLac2*

**Fig. 1:**
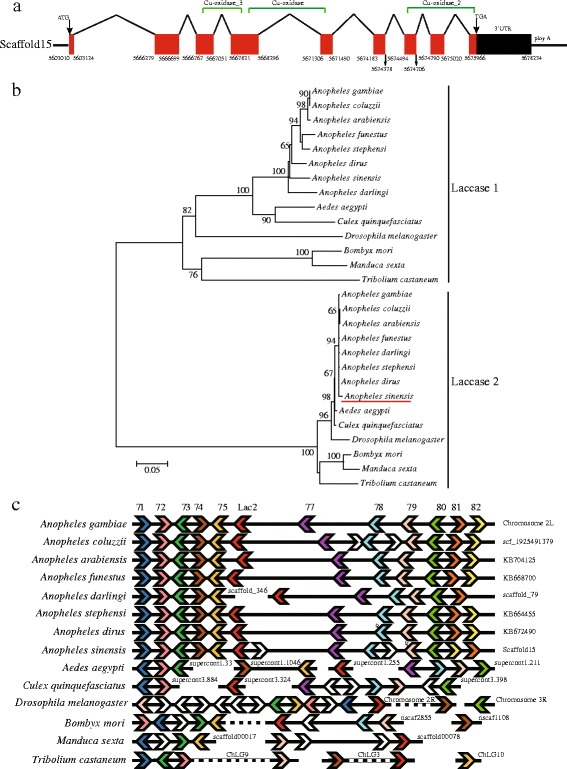
Gene structure of *AsLac2*, phylogenetic analysis of Laccase 1 (LAC1) and Laccase 2 (LAC2) proteins among representative insect species, and genome synteny of the neighborhood of *Laccase 2*. **a** Solid red boxes represent amino acid coding sequences and fold lines represent the introns. Solid black box represents the 3′UTR. Green square brackets (horizontal) represent the Cu-oxidase domains. **b** Maximum likelihood phylogenetic tree of Laccase 1 and Laccase 2 among different insects. Amino acid sequence information are as follow: *Anopheles gambiae* (LAC1: AGAP003738-PA; LAC2: AGAP006176-PB), *Anopheles coluzzii* (LAC1: ACOM034942-PA; LAC2: ACOM024569-PA), *Anopheles arabiensis* (LAC1: AARA010056-PA; LAC2: AARA007346-PA BAG70891), *Anopheles funestus* (LAC1: AFUN001510-PA; LAC2: AFUN004369-PA), *Anopheles stephensi* (LAC1: ASTE005343-PA; LAC2: ASTE006211-PA), *Anopheles dirus* (LAC1: ADIR002561-PA; LAC2: ADIR007990-PA), *Anopheles darling* (LAC1: ADAC007761-PA; LAC2: ADAC006306 -PA), *Aedes aegypti* (LAC1: AAEL007802 -PA; LAC2: AAEL007415-PA), *Culex quinquefasciatus* (LAC1: CPIJ012357-PA; LAC2: CPIJ010466-PA), *Bombyx mori* (LAC1: XP_012552135; LAC2: BAG70891), *Manduca sexta* (LAC1: AY135185; LAC2: AAN17507), *Drosophila melanogaster* (LAC1: NP_609287; LAC2: NP_724412), and *Tribolium castaneum* (LAC1: NP_001034514; LAC2: NP_001034487); **c** Genomic or scaffold synteny analysis of the neighborhood of *Laccase 2* in different insects using the *Anopheles gambiae* genome as the reference. Solid horizontal lines represent the chromosome or scaffold. Orthologue genes are represented by the swallowtail symbols with the same colour (numbers represent gene IDs (The prefix is AGAP0061)). The small triangles linked with the swallowtail represent intron-located genes. The tip of the swallowtail symbols indicates the direction of transcription. Black and white interval lines represent the insertion of multiple genes

The *Anopheles sinensis Laccase 2* (*AsLac2*) is 4,391 bp (without poly A) with a 2,265 bp ORF encoding 754 amino acids, and a 2,126 bp 3′UTR (GenBank Number: KY132102); a 26-residue signal peptide was predicted, suggesting it is a secreted protein. Conserved domain prediction revealed that *AsLac2* contains three domains: Cu-oxidase3, Cu-oxidase, and Cu-oxidase 2 (Fig. 1a).

Phylogenetic analysis showed that representative insect Laccases were grouped into two distinct clades (Fig. 1b), Laccase 1 and Laccase 2. The newly cloned *Laccase* gene was identified as a *Laccase 2* homologue. Three Cu-oxidase domains for typical Laccase 2 are highly conserved in different insects and exhibit 81.6 to 100% identities (Additional file 2: Table S2). In particularly, within the genus *Anopheles*, the identities range from 92.3% to 100% for three Cu-oxidase domains (Additional file 2: Table S2). These features suggest that their functions are likely conserved in different insect species. Moreover, chromosomal or scaffold synteny analyses revealed the neighborhood genes around *Laccase 2* are more conservative across the genus *Anopheles* than in other insect species (Fig. 1c), which may imply the conservation of this genome fragment during the evolution and divergence of mosquitoes. Further analyses found that upstream regulatory elements of *Lac2*, like ‘BR-C’, ‘E74’, ‘FTZ’, and ‘ECR::USP’, occurs in all analyzed *Lac2* (Additional file 3: Table S3), suggesting that *Lac2* is regulated by ecdysone during development [47–49].

### The *AsLaccase 2* expression pattern is in good agreement with pupal cuticle tanning degree

**Fig. 2:**
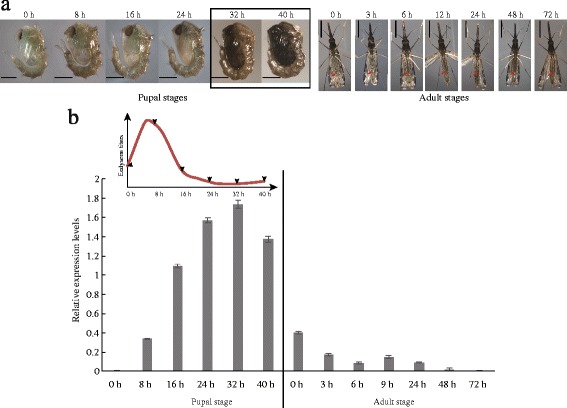
Association between phenotype and *AsLac2* expression during the pupal-adult developmental stages. **a** Phenotypes of female *Anopheles sinensis* at different development stages (from pupae to early adults). The black box represents the stages of rapid melanism. The red arrows indicate the melanism of the adult abdomen (The melanin degree and changes are not as dramatic as the pupal stages). **b** Expression pattern of *AsLac2* from the pupal stages to the early adult stages as well as the derived ecdysone titers in the pupal stage are cited from [47]. *AsRPL49* was used as the internal control. *Scale-bars*: 500 μm

Both male and female *Anopheles sinensis* reared in our laboratory required about 40–42 h to complete the eclosion. According to our observations of pupae phenotype in different developmental stages, the cuticle tanning pattern is the same in the male and female pupae [12]. The cuticle colour of the female pupae is a little transparent with a slight yellowish tint within 24 h after pupation (Fig. 2a) and subsequently, strong melanization and hardening were observed until the late pupal stage, especially at 32 h after pupation (Fig. 2a). Therefore, we concluded that the expression levels of key genes involved in melanin synthesis and cuticle tanning should be significantly increased with the tanning degrees. The expression of *A*s*Lac2* was almost undetectable at 0 h after pupation, while its expression started to be upregulated at 8 h, was remarkably increased at 16 h, and was maintained to 40 h after pupation (Fig. 2b). This expression pattern positively correlated well with the cuticle melanization and sclerotization process, but negatively correlated with the derived hemolymph ecdysone titer during the mosquito pupal stage [47] (Fig. 2b). Interestingly, a retarded expression pattern of *AsLac2* was observed when compared with the upstream, rate-limited gene *AsTH* [12], indicating that the catalytic function of *AsLac2* occurred after the accumulation of catecholamine. Furthermore, we found that the expression intensity of *AsLac2* in the pupae (from the early-middle to the last stage) was significantly stronger than that in the adults (Fig. 2a), implying its importance in normal pupal development (Fig. 2). Therefore, we concluded that *Aslac2* is closely linked to pupae cuticle tanning.

### *AsLaccase 2* depletion results in pupal cuticle tanning defects and eclosion retardation

**Fig. 3:**
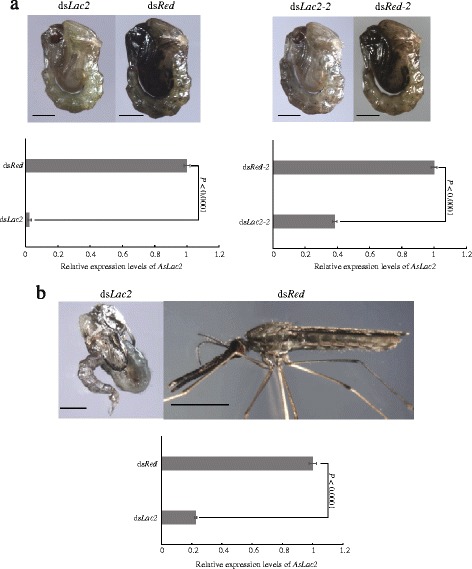
Effects of silencing *AsLac2* on pupa and adult cuticle tanning. **a** Effects of two non-overlapping ds*Lac2* on pupal cuticle tanning at 38 h after the pupation (unpaired *t*-test: *t*_(4)_ = 78.86, *P* < 0.0001 for ds*Lac2*; *t*_(4)_ = 47.87, *P* < 0.0001 for ds*Lac2-2*; *n* = 3). **b** The molting and cuticle tanning in *AsLac2*-silenced, newly emerged adult (3 h after emergence, unpaired *t*-test: *t*_(4)_ = 51.84, *P* < 0.0001, *n* = 3)*. AsRPL49* gene was used as the internal control. *Scale-bars*: 500 μm

**Fig. 4:**
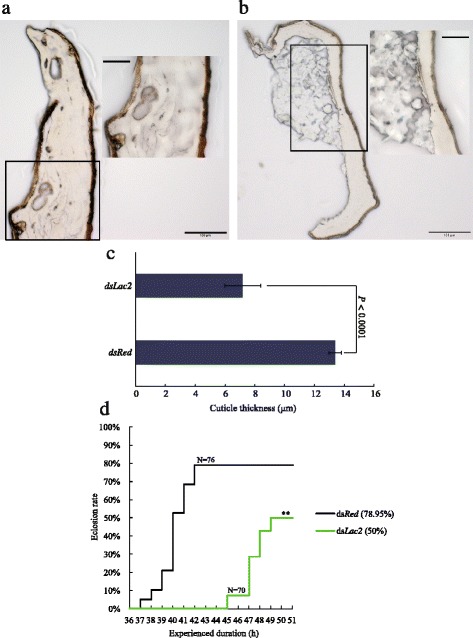
Effects of silencing *AsLac2* on physical characteristics of the adult cuticle and emergence rate. Observation and thickness measurement of the dorsal plate cuticle of individual adults in ds*Lac2-*injected (**b**) and ds*Red*-injected groups (**a**). Insets represent enlarged images from the boxed region. **c** Graphical representation of cuticle thickness for the two groups. An unpaired *t*-test was performed to test the difference in cuticle thickness between the two groups (*t*_(6)_ = 9.67, *P <* 0.0001, *n* = 4). **d** Comparison of emergence time and emergence rate between *dsLac2-* and ds*Red*-injected groups (N, represents the sample size; log rank test, **P < 0.01). *Scale-bars*: a, b, 100 μm; insets, 50 μm

Normally, the pupal cuticle darkens and gradually hardens within 24 h of development, and becomes much darker at 38 h after pupation. In this study, no pigmentation blocking was observed in the ds*Red*-injected individuals as they exhibited a normal development and tanning process (Fig. 3a). In contrast, ds*Lac2*-injected pupae exhibited very little or even no melanism at 38 h after pupation, and were accompanied by very soft cuticles (Fig. 3a). At this time point, the expression level of *AsLac2* was much lower than that in the ds*Red-*injected group (Fig. 3a). To avoid the off-target effect, we designed another pair of primers (ds*Las2-*2) without overlapping with ds*Lac*2. There were 80 and 66% of individuals whose cuticle was not tanned and flexible that were obtained in the ds*Lac2* and ds*Lac2-2* groups, respectively (Fig. 3a, Additional file 4: Table S4). In the ds*Red*-injected group, the cephalothorax was almost completely tanned at 3 h after adult emergence (Fig. 3b); however, the ds*Lac2*-injected pupae were extremely struggling to emerge and the adults displayed a very light body colour and soft cephalothorax (Fig. 3b). Moreover, we found that the thickness of the dorsal plate section of the cephalothorax in the ds*Lac2*-injected pupae (very fragile and easy to break) (Fig. 4b) was only about 54% (Fig. 4c) of that in the control group (Fig. 4a). The expression level of ds*Lac2* was also significantly lower than in the control group (Fig. 3b). Our present data indicates that *AsLac2* dysfunction can lead to cuticle defects in the melanizaiton and sclerotization processes, suggesting that it is crucial for cuticle tanning (Fig. 3, Fig. 4a, b and c). In the control group, the pupae started to emerge at 37 h and completed the pupation at 41 h with the eclosion rate reaching 79% (Fig. 4d). However, only a 50% eclosion rate was obtained from 45 h to 49 h after pupation in the *dsLac2*-injected group (Fig. 4d). Obviously, there was an 8-h eclosion retardation in the ds*Lac2*-injected groups (Fig. 4d). Some of the remaining pupae barely emerged, were physically weak and had wing vibration defects, which distinctly impaired the adults’ ability to survive. The obvious time lag, low emergence rate, and physical weakness definitely affected the mosquito population.

### Dysfunctional *AsLaccase 2* caused the resistance of pathogen reducedsharply in the pupae

**Fig. 5:**
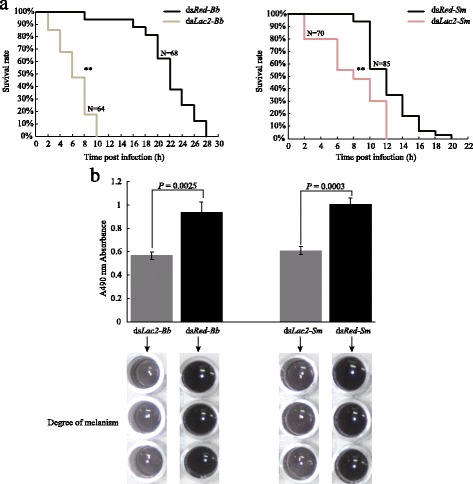
Comparison of exogenous pathogen resistance in the ds*Lac2-* and the ds*Red*-injected pupae. **a** Survival curve of the ds*Lac2-* and the ds*Red*-injected pupae infected by *B. bombyseptie* (*Bb*) or *S. marcescens* (*Sm*) (N represent the sample size; log rank test, *P* < 0.01). **b** Comparison of in vivo melaninization degree in ds*Lac2*- and ds*Red*-injected pupae infected by *Bb* or *sm* (unpaired *t*-test: *t*_(4)_ = 6.72, *P* = 0.0025 for *Bb* infected group, *n* = 3; *t*_(4)_ = 11.89*, P =* 0.0003 for *Sm* infected group, *n* = 3)

In the ds*Lac2*-injected group, pupa death started at 2 h after *Bb* challenge, then the mortality increased with the time going from 2 to 10 h and all individuals died at 10 h after the injection (Fig. 5a). In contract, large-scale mortality in the ds*Red* group occurred at 20–24 h after the bacteria challenge, which was significantly delayed compared to those in the ds*Lac2*-injected group (*P* < 0.01) (Fig. 5a). Although, the mortality rate in the *dsRed-* and *dsLac2*-injected groups caused by *Sm* was not significantly more severe that those infected by *Bb*, the survival and life span of the *dsLac2*-injected pupae were still obviously decreased by *Sm* (*P* < 0.01) (Fig. 5a). The in vivo assay results revealed that the melanization degree of ds*Lac2-*injected pupal humor was significantly lower than those in the controls after *Bb* or *Sm* infection, suggesting that the melanization immune response was severely attenuated in the *dsLac2-*injected pupae (Fig. 5b). In conclusion, it is worth noting that *dsLac2-*injected pupae died rapidly and severely after *Bb* or *Sm* infections (Fig. 5). These results verified that the *AsLac2* gene is involved in *Anopheles sinensis*’ innate immunity. *AsLac2* dysfunction greatly suppressed the melanization immune responses to bacterial infections, which further caused quick and severe death.

## Discussion

Laccase 2 is mainly synthesized in epidermis cells and secreted to the procuticle [5, 34, 38, 40–44]. Subsequently, it catalyzes the oxidation of catecholamine to quinones and/or quinone methides, which are involved in cuticle pigmentation, as well as stabilizes and reinforces the cuticle structure *via* catalyzing the cross-linking among various cuticular components, such as proteins-proteins, proteins-melanin, or proteins-other quinones and/or quinone methides [3–5, 44, 50]. This implies that its normal function controls the supply of important materials for cuticle construction and maintenance. Therefore, the expression pattern of *Laccase 2* in a specific developmental stage should reflect the characteristics and changes of the cuticle. In the present study, the expression of *AsLac2* was significantly upregulated in the middle and the late pupal stage (Fig. 2). The good agreement of this expression pattern and rapid cuticle tanning strongly supports this view. Serious defects in the pupal cuticle caused by *AsLac2* silencing in the high expression period undoubtedly imply that it plays a decisive role in the maintenance of cuticle integrity during mosquito pupal metamorphosis.

Moreover, in most insects, *Laccase 2* is irreplaceable and dominantly expressed in pupae, while other melanin metabolism genes, such as *TH* or *Yellow* [38, 40, 51–53], are not always dominantly expressed in the pupal stage. Therefore, we thought that the pupae could protect itself from external adverse factors mainly through passive defenses during pupal and/or eclosion periods. The high expression of *Laccase 2* in this stage can promote quinone and/or quinonemethide accumulation to enhance, harden, and steady the pupal cuticle (Figs. 2, 3 and 4). It is also beneficial for adult cuticle formation to adapt to the environment. On the other hand, ecdysone titer peaks during the early and the middle pupa developmental stage further trigger apolysis and promote the formation of the new pupal cuticle [47–49]. The upregulation of *AsLac2* in this period verifies the general rule that the formation of the new cuticle and the expression patterns of *Laccase 2* are modulated by the ecdysone titer and their functional conservations in the insect evolutionary processes.

Two alternative isoforms of *Laccase 2*, *Lac2A* and *Lac2B*, were identified in some insects [34, 38]. They share the same N-terminal structure and three Cu-oxidase domains, but the C-terminals are quite divergent. Previous studies showed that *Lac2A* was definitely up-regulated in the cuticle, but not *Lac2B* [34, 38]. Although the expression pattern and abundance were different in vivo, no significant differences in substrate preferences were observed between the recombinant Lac2A and Lac2B [54]. In this study, two alternative splicing forms of *Laccase 2* were also predicted in the *Anopheles sinensis* genome (Additional file 5: Figure S1; Additional file 6: Dataset 1). The phylogenetic analysis revealed that insect LAC2s were grouped into two distinct clads (Additional file 5: Figure S1). Lac2As are highly conserved with high bootstrap supports, but Lac2Bs are quite divergent (Additional file 5: Figure S1). This result implies that Lac2B may be more diverse in functions. However, no transcripts of *AsLac2B* were detected in our analysis; this may be because their expression is undetectable or they are dominantly expressed in other development stages or tissues. Although the dsRNA fragment was designed according to the common region of *Lac2A* and predicted *Lac2B*, no expression of *Lac2B* was detected in the high expression period and the tissues selected for *Lac2A* silencing. Moreover, the defective phenotype caused by RNAi was due to the dramatic downregulation of *Lac2A* expression, which is in good agreement with previous reports [38, 40–42, 44]. Therefore, we concluded that the defective phenotype was caused by *Lac2A*, but not *Lac2B*. Further studies will be conducted to investigate the expression pattern of *Lac2B* in other development stages (such as the adult stages) and more fine tissues, which can further help to understand its functions.

Melanin generated from the precursors of dopa and dopamine by the activated phenol oxidases (POs) is important for immune responses [5, 7–9, 12, 14–16, 19, 23, 24, 55, 56]. Genes (*TH*, *DDC* etc*.*) required for melanin synthesis are distributed in immune tissues including fat body and hemolymph [12, 57, 58]. Thus, immune tissues can utilize these precursors in melanization immune reactions catalyzed by phenol oxidases, such as tyrosinase or prophenoloxidases. These reactions are crucial for pathogen resistance. Laccase 2, a member of the phenol oxidase family, can catalyze the precursors to form melanin. If this gene can be expressed in some other immune tissues besides the cuticle of invertebrates [31, 34], theoretically, it can catalyze the terminal oxidation reaction for melanin synthesis. Our results revealed that *AsLac2* was expressed in the pupal immune tissues (Additional file 7: Figure S2); this is similar to *Laccase 2* expression being slightly increased in *Anopheles gambiae* after bacterial infection [34]. Combined with our results showing that the melanization in the *AsLac2*-silenced pupae was significantly impaired and the resistance to bacteria challenges was dramatically decreased (Fig. 5), we notice the close relationship between the expression of *AsLac2* and melanization immune responses. We speculate that *AsLac2* may exert prophenoloxidase activity and involve melanotic immune responses. The dysfunction of *AsLac2* can result in insufficient melanin synthesis and further impair immune responses to bacterial pathogens. Additionally, it is unclear whether immune responses are impaired by the feedback inhibition to catecholamine synthase caused by the temporarily accumulation of catecholamine, which is due to the silencing of *AsLac2*. This feedback inhibition may reduce the efficiency of melanin metabolism, in which the formation of the melanin precursors is further affected. To test this hypothesis, in vitro assays will be conducted to test the activity of total phenoloxidases in melanization and to determine the catecholamine content in *dsLac2*-injected pupae in subsequent research.

## Conclusions

To our knowledge, the present results are the first to illustrate that the suppression of Laccase 2 results in serious adverse effects, which are almost lethal for the wild mosquitoes during the pupal development of *Anopheles sinensis*. As a rate-limiting enzyme in the final step of melanin metabolism, *Laccase 2* has a similar expression pattern as the initial rate-limiting enzyme gene *TH* in *Anopheles sinensis* [12]. Silencing these two genes produced similar physiological defective phenotypes, further suggesting that *Laccase 2* and *TH* are crucial for normal pupal development. Our studies further corroborated that melanin metabolism is indispensable for the normal development of mosquito pupae. Therefore, the key genes and/or their regulatory elements in the melanin metabolism pathway may be valuable targets for mosquito prevention and control.

## Additional files

Additional file 1: Table S1. Primers used in this study. (XLSX 11 kb)

Additional file 2: Table S2. Amino acid sequence identity of Cu-oxidase domains of LAC2 orthologs. (PDF 109 kb)

Additional file 3: Table S3. Prediction of *cis*-acting transcriptional regulatory elements upstream. 7.5 kb of *Laccase 2*. (XLSX 9 kb)

Additional file 4: Table S4. Statistical analysis of pupal cuticle tanning degree in the RNAi experiment at 38 h after pupation. (DOC 28 kb)

Additional file 5: Figure S1. Alternative splicing and amino acid sequence analyses of Laccase 2 in representative insect species. **a** Predicted alternative splicing forms of *AsLac2*. Red and green boxes represent the special exon of Laccase 2A and Laccase 2B, respectively. **b** Maximum Likelihood phylogenetic tree of two Laccase 2 forms in different insect species. **c** Genetic distance estimations among LAC2As (red) and LAC2Bs (yellow). (PDF 97 kb)

Additional file 6: Dataset 1. The identified Laccase 2A and the predicted Laccase 2B isoform in *Anopheles sinensis*. (DOCX 15 kb)

Additional file 7: Figure S2. Gene expression patterns of *AsLac2* in pupal cuticle, fat body, and hemolymph. *AsRPL49* was used as the internal control. (PDF 72 kb)

## Abbreviations

*AsLac2*: *Anopheles sinensis Laccase 2*
*Bb*: *Bacillus bombyseptie*
DDC: DOPA decarboxylase
dopa: Dihydroxy phenylalanine
LAC1: Laccase 1
LAC2: Laccase 2
POs: phenol oxidases
*Sm*: *Serratia marcescens*
TH: tyrosine hydroxylase

## References

[1] Sugumaran M. Unified mechanism for sclerotization of insect cuticle. Adv Insect Physiol. 1998;27:229–334.

[2] Sugumaran M. Chemistry of cuticular sclerotization. Adv Insect Physiol. 2010;39:151.

[3] Andersen SO (2010). Insect cuticular sclerotization: a review. Insect Biochem Mol Biol.

[4] Hopkins TL, Kramer KJ (1992). Insect cuticle sclerotization. Annu Rev Entomol.

[5] Sugumaran M, Barek H (2016). Critical analysis of the melanogenic pathway in insects and higher animals. Int J Mol Sci.

[6] Vincent J, Hillerton J. The tanning of insect cuticle - a critical review and a revised mechanism. J Insect Physiol. 1979;25(8):653–8.

[7] Hultmark D (1993). Immune reactions in *Drosophila* and other insects: a model for innate immunity. Trends Genet.

[8] Sugumaran M (2002). Comparative biochemistry of eumelanogenesis and the protective roles of phenoloxidase and melanin in insects. Pigment Cell Res.

[9] Schmid-Hempel P (2005). Evolutionary ecology of insect immune defenses. Annu Rev Entomol.

[10] Caraballo H, King K (2014). Emergency department management of mosquito-borne illness: malaria, dengue, and West Nile virus. Emerg Med Pract.

[11] Tolle MA (2009). Mosquito-borne diseases. Curr Probl Pediatr Adolesc Health Care.

[12] Qiao L, Du M, Liang X, Hao Y, He X, Si F (2016). Tyrosine hydroxylase is crucial for maintaining pupal tanning and immunity in *Anopheles sinensis*. Sci Rep.

[13] Johnson JK, Rocheleau TA, Hillyer JF, Chen CC, Li J, Christensen BM (2003). A potential role for phenylalanine hydroxylase in mosquito immune responses. Insect Biochem Mol Biol.

[14] Christensen BM, Li J, Chen CC, Nappi AJ (2005). Melanization immune responses in mosquito vectors. Trends Parasitol.

[15] Wilson K, Cotter SC, Reeson AF, Pell JK (2001). Melanism and disease resistance in insects. Ecol Lett.

[16] Osta MA, Christophides GK, Vlachou D, Kafatos FC (2004). Innate immunity in the malaria vector *Anopheles gambiae*: comparative and functional genomics. J Exp Biol.

[17] Kramer KJ, Hopkins TL (1987). Tyrosine metabolism for insect cuticle tanning. Arch Insect Biochem Physiol.

[18] True JR (2003). Insect melanism: the molecules matter. Trends Ecol Evol.

[19] Kanost MR, Jiang H, Yu XQ. Innate immune responses of a lepidopteran insect, *Manduca sexta*. Immunol Rev. 2004;198:97–105. 10.1111/j.0105-2896.2004.0121.x 15199957

[20] Prota G (1992). Melanins and melanogenesis.

[21] Ito S (2003). A chemist’s view of melanogenesis. Pigment Cell Res.

[22] Sugumaran M, Duggaraju R, Generozova F. Insect melanogenesis. II. Inability of *Manduca* phenoloxidase to Act on 5, 6-dihydroxyindole-2-carboxylic acid1. Pigment Cell Res. 1999;12(2):118–25. 10.1111/j.1600-0749.1999.tb00751.x 10231199

[23] Shao Q, Yang B, Xu Q, Li X, Lu Z, Wang C (2012). Hindgut innate immunity and regulation of fecal microbiota through melanization in insects. J Biol Chem.

[24] Soderhall K, Cerenius L (1998). Role of the prophenoloxidase-activating system in invertebrate immunity. Curr Opin Immunol.

[25] Cerenius L, Lee BL, Soderhall K (2008). The proPO-system: pros and cons for its role in invertebrate immunity. Trends Immunol.

[26] Zhukhlistova N, Zhukova YN, Lyashenko A, Zaĭtsev V, Mikhaĭlov A (2008). Three-dimensional organization of three-domain copper oxidases: a review. Crystallogr. Rep..

[27] Mayer AM, Staples RC (2002). Laccase: new functions for an old enzyme. Phytochemistry.

[28] Sakurai T, Kataoka K (2007). Basic and applied features of multicopper oxidases, CueO, bilirubin oxidase, and laccase. Chem Rec.

[29] Dittmer NT, Gorman MJ, Kanost MR. Characterization of endogenous and recombinant forms of laccase-2, a multicopper oxidase from the tobacco hornworm, *Manduca sexta*. Insect Biochem Mol Biol. 2009;39(9):596–606. 10.1016/j.ibmb.2009.06.006 PMC2733336 19576986

[30] Dittmer NT, Kanost MR (2010). Insect multicopper oxidases: diversity, properties, and physiological roles. Insect Biochem Mol Biol.

[31] Dittmer NT, Suderman RJ, Jiang H, Zhu YC, Gorman MJ, Kramer KJ, et al. Characterization of cDNAs encoding putative laccase-like multicopper oxidases and developmental expression in the tobacco hornworm, *Manduca sexta*, and the malaria mosquito, *Anopheles gambiae*. Insect Biochem Mol Biol. 2004;34(1):29–41. 10.1016/j.ibmb.2003.08.003 14723895

[32] Hattori M, Tsuchihara K, Noda H, Konishi H, Tamura Y, Shinoda T (2010). Molecular characterization and expression of laccase genes in the salivary glands of the green rice leafhopper, *Nephotettix cincticeps* (Hemiptera: Cicadellidae). Insect Biochem Mol Biol.

[33] Yatsu J, Asano T (2009). Cuticle laccase of the silkworm, *Bombyx mori*: purification, gene identification and presence of its inactive precursor in the cuticle. Insect Biochem Mol Biol.

[34] Gorman MJ, Dittmer NT, Marshall JL, Kanost MR (2008). Characterization of the multicopper oxidase gene family in *Anopheles gambiae*. Insect Biochem Mol Biol.

[35] Asano T, Taoka M, Yamauchi Y, Everroad RC, Seto Y, Isobe T (2014). Re-examination of a α-chymotrypsin-solubilized laccase in the pupal cuticle of the silkworm, *Bombyx mori*: Insights into the regulation system for laccase activation during the ecdysis process. Insect Biochem Mol Biol.

[36] Lang M, Kanost MR, Gorman MJ (2012). Multicopper oxidase-3 is a laccase associated with the peritrophic matrix of *Anopheles gambiae*. PLoS One.

[37] Thomas B, Yonekura M, Morgan T, Czapla T, Hopkins T, Kramer K (1989). A trypsin-solubilized laccase from pharate pupal integument of the tobacco hornworm, Manduca sexta. Insect Biochem.

[38] Arakane Y, Muthukrishnan S, Beeman RW, Kanost MR, Kramer KJ. Laccase 2 is the phenoloxidase gene required for beetle cuticle tanning. Proc Natl Acad Sci USA. 2005;102(32):11337–42. 10.1073/pnas.0504982102 PMC1183588 16076951

[39] Wu X, Zhan X, Gan M, Zhang D, Zhang M, Zheng X (2013). Laccase2 is required for sclerotization and pigmentation of *Aedes albopictus* eggshell. Parasitol Res.

[40] Elias-Neto M, Soares MP, Simoes ZL, Hartfelder K, Bitondi MM (2010). Developmental characterization, function and regulation of a Laccase2 encoding gene in the honey bee, *Apis mellifera* (Hymenoptera, Apinae). Insect Biochem Mol Biol.

[41] Niu BL, Shen WF, Liu Y, Weng HB, He LH, Mu JJ (2008). Cloning and RNAi-mediated functional characterization of MaLac2 of the pine sawyer, Monochamus alternatus. Insect Mol Biol.

[42] Futahashi R, Tanaka K, Matsuura Y, Tanahashi M, Kikuchi Y, Fukatsu T (2011). Laccase2 is required for cuticular pigmentation in stinkbugs. Insect Biochem Mol Biol.

[43] Futahashi R, Banno Y, Fujiwara H (2010). Caterpillar color patterns are determined by a two-phase melanin gene prepatterning process: new evidence from tan and laccase2. Evol Dev.

[44] Masuoka Y, Miyazaki S, Saiki R, Tsuchida T, Maekawa K (2013). High Laccase2 expression is likely involved in the formation of specific cuticular structures during soldier differentiation of the termite *Reticulitermes speratus*. Arthropod Struct Dev.

[45] Chen B, Zhang YJ, He Z, Li W, Si F, Tang Y (2014). *De novo* transcriptome sequencing and sequence analysis of the malaria vector *Anopheles sinensis* (Diptera: Culicidae). Parasit Vectors.

[46] Tamura K, Peterson D, Peterson N, Stecher G, Nei M, Kumar S (2011). MEGA5: molecular evolutionary genetics analysis using maximum likelihood, evolutionary distance, and maximum parsimony methods. Mol Biol Evol.

[47] Margam VM, Gelman DB, Palli SR (2006). Ecdysteroid titers and developmental expression of ecdysteroid-regulated genes during metamorphosis of the yellow fever mosquito, *Aedes aegypti* (Diptera: Culicidae). J Insect Physiol.

[48] Thummel CS (2001). Molecular mechanisms of developmental timing in *C. elegans* and *Drosophila*. Dev Cell.

[49] Dubrovsky EB (2005). Hormonal cross talk in insect development. Trends Endocrinol Metab.

[50] Mun S, Noh MY, Dittmer NT, Muthukrishnan S, Kramer KJ, Kanost MR (2015). Cuticular protein with a low complexity sequence becomes cross-linked during insect cuticle sclerotization and is required for the adult molt. Sci Rep.

[51] Ito K, Yoshikawa M, Fujii T, Tabunoki H, Yokoyama T (2016). Melanin pigmentation gives rise to black spots on the wings of the silkworm *Bombyx mori*. J Insect Physiol.

[52] Liu C, Yamamoto K, Cheng TC, Kadono-Okuda K, Narukawa J, Liu SP, et al. Repression of tyrosine hydroxylase is responsible for the sex-linked chocolate mutation of the silkworm, *Bombyx mori*. Proc Natl Acad Sci USA. 2010;107(29):12980–5. 10.1073/pnas.1001725107 PMC2919899 20615980

[53] Futahashi R, Sato J, Meng Y, Okamoto S, Daimon T, Yamamoto K (2008). Yellow and ebony are the responsible genes for the larval color mutants of the silkworm *Bombyx mori*. Genetics.

[54] Gorman MJ, Sullivan LI, Nguyen TD, Dai H, Arakane Y, Dittmer NT (2012). Kinetic properties of alternatively spliced isoforms of laccase-2 from *Tribolium castaneum* and *Anopheles gambiae*. Insect Biochem Mol Biol.

[55] Dubovskiy IM, Whitten MM, Kryukov VY, Yaroslavtseva ON, Grizanova EV, Greig C, et al. More than a colour change: insect melanism, disease resistance and fecundity. Proc Biol Sci. 2013;280(1763):20130584. 10.1098/rspb.2013.0584 PMC3774225 23698007

[56] Eleftherianos I, Revenis C (2011). Role and importance of phenoloxidase in insect hemostasis. J Innate Immun.

[57] Huang CY, Chou SY, Bartholomay L, Christensen B, Chen CC (2005). The use of gene silencing to study the role of dopa decarboxylase in mosquito melanization reactions. Insect Mol Biol.

[58] Sideri M, Tsakas S, Markoutsa E, Lampropoulou M, Marmaras VJ (2008). Innate immunity in insects: surface-associated dopa decarboxylase-dependent pathways regulate phagocytosis, nodulation and melanization in medfly haemocytes. Immunology.

