# Supplementary material for: Suppression of Laccase 2 severely impairs cuticle tanning and pathogen resistance during the pupal metamorphosis of Anopheles sinensis (Diptera: Culicidae)
Source: Parasit Vectors. 2017 Apr 4;10:171. doi: 10.1186/s13071-017-2118-4 (PMC5381134; doi:10.1186/s13071-017-2118-4)
Supplement: Supplementary file 2 — Amino acid sequence identity of Cu-oxidase domains of LAC2 orthologs. (PDF 109 kb) [file 13071_2017_2118_MOESM2_ESM.pdf]

Additional file 2

	Bombyx mori	Manduca sexta	Tribolium castaneum	Drosophila melanogaster	aedes aegypti	Culex quinquefasciatus	Anopheles sinensis	Anopheles dirus A	Anopheles arabiensis	Anopheles funestus	Anopheles darlingi	Anopheles stephensi	Anopheles coluzzii	Anopheles gambiae
Bombyx mori	–													
Manduca sexta	0.956	–												
Tribolium castaneum	0.844	0.836	–											
Drosophila melanogaster	0.87	0.855	0.855	–										
aedes aegypti	0.831	0.821	0.816	0.876	–									
Culex quinquefasciatus	0.865	0.858	0.852	0.936	0.905	–								
Anopheles sinensis	0.87	0.863	0.848	0.929	0.903	0.978	–							
Anopheles dirus A	0.867	0.86	0.85	0.936	0.91	0.982	0.988	–						
Anopheles arabiensis	0.867	0.86	0.85	0.934	0.907	0.98	0.99	0.995	–					
Anopheles funestus	0.867	0.86	0.848	0.934	0.908	0.98	0.99	0.996	0.995	–				
Anopheles darlingi	0.831	0.823	0.822	0.889	0.964	0.923	0.923	0.931	0.928	0.928	–			
Anopheles stephensi	0.829	0.821	0.821	0.885	0.959	0.92	0.923	0.928	0.928	0.928	0.995	–		
Anopheles coluzzii	0.831	0.823	0.819	0.889	0.957	0.921	0.925	0.929	0.929	0.929	0.991	0.996	–	
Anopheles gambiae	0.831	0.823	0.819	0.889	0.957	0.921	0.925	0.929	0.929	0.929	0.991	0.996	1	–
