# Supplementary material for: Suppression of Laccase 2 severely impairs cuticle tanning and pathogen resistance during the pupal metamorphosis of Anopheles sinensis (Diptera: Culicidae)
Source: Parasit Vectors. 2017 Apr 4;10:171. doi: 10.1186/s13071-017-2118-4 (PMC5381134; doi:10.1186/s13071-017-2118-4)
Supplement: Supplementary file 4 — Statistical analysis of pupal cuticle tanning degree in the RNAi experiment at 38 h after pupation. (DOC 28 kb) [file 13071_2017_2118_MOESM4_ESM.doc]

Additional file 4

| Treatment | Injected No. | No. with tanning impaired | No. of Death |
| --- | --- | --- | --- |
| ds*Lac2* | 29 | 21 | 3 |
| ds*Lac2-2* | 32 | 20 | 2 |
| ds*Red* (for ds*Lac2*) | 30 | 0 | 1 |
| ds*Red* (for ds*Lac2*-2) | 27 | 0 | 2 |
