# Supplementary material for: Suppression of Laccase 2 severely impairs cuticle tanning and pathogen resistance during the pupal metamorphosis of Anopheles sinensis (Diptera: Culicidae)
Source: Parasit Vectors. 2017 Apr 4;10:171. doi: 10.1186/s13071-017-2118-4 (PMC5381134; doi:10.1186/s13071-017-2118-4)
Supplement: Supplementary file 5 — Alternative splicing and amino acid sequence analyses of Laccase 2 in representative insect species. a Predicted alternative splicing forms of AsLac2. Red and green boxes represent the special exon of Laccase 2A and Laccase 2B, respectively. b Maximum Likelihood phylogenetic tree of two Laccase 2 forms in different insect species. c Genetic distance estimations among LAC2As (red) and LAC2Bs (yellow). (PDF 97 kb) [file 13071_2017_2118_MOESM5_ESM.pdf]

Additional file 5

a

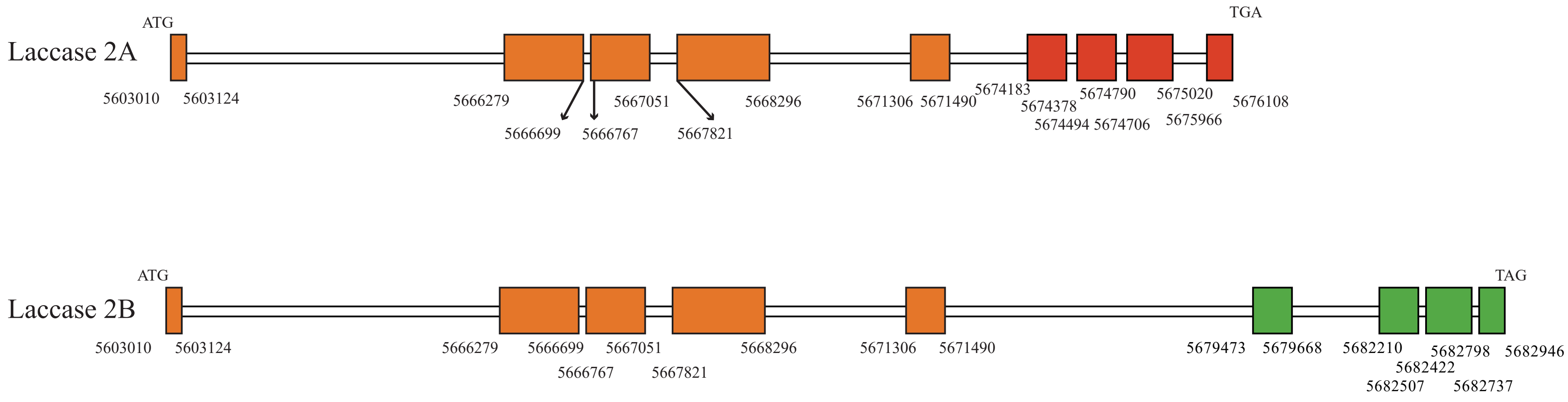

b

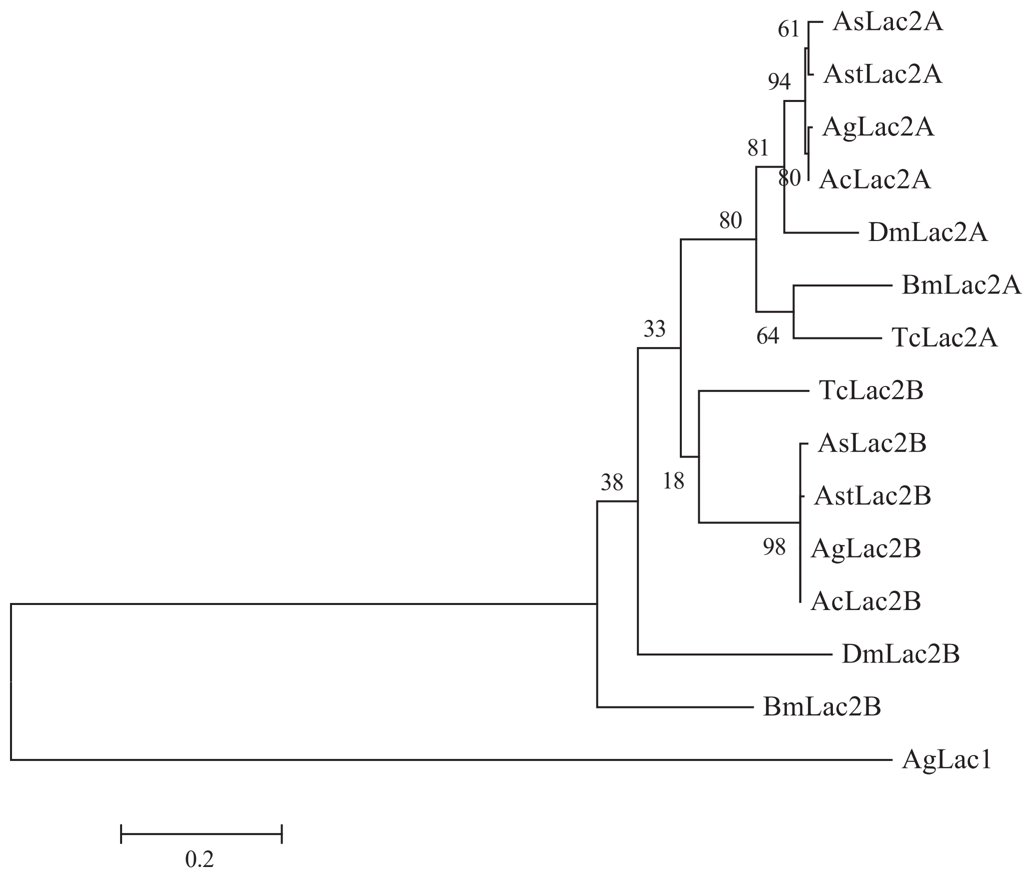

c

	AgLac1	BmLac2B	DmLac2B	TcLac2B	AsLac2B	AstLac2B	AgLac2B	AcLac2B	BmLac2A	TcLac2A	DmLac2A	AsLac2A	AstLac2A	AgLac2A	AcLac2A
AgLac1															
BmLac2B	1.336														
DmLac2B	1.335	0.396													
TcLac2B	1.276	0.32	0.338												
AsLac2B	1.292	0.38	0.294	0.233											
AstLac2B	1.303	0.375	0.291	0.228	0.012										
AgLac2B	1.28	0.38	0.293	0.235	0.008	0.004									
AcLac2B	1.288	0.371	0.286	0.229	0.008	0.004	0								
BmLac2A	1.159	0.372	0.394	0.274	0.323	0.33	0.334	0.326							
TcLac2A	1.236	0.387	0.372	0.268	0.279	0.286	0.293	0.286	0.19						
DmLac2A	1.307	0.397	0.347	0.276	0.271	0.282	0.28	0.273	0.204	0.185					
AsLac2A	1.299	0.34	0.339	0.254	0.233	0.24	0.241	0.235	0.179	0.184	0.117				
AstLac2A	1.29	0.339	0.34	0.241	0.221	0.227	0.228	0.223	0.183	0.179	0.108	0.02			
AgLac2A	1.253	0.348	0.34	0.244	0.227	0.234	0.232	0.232	0.193	0.179	0.115	0.025	0.016		
AcLac2A	1.269	0.335	0.337	0.243	0.217	0.224	0.227	0.222	0.184	0.17	0.108	0.02	0.012	0.004	
