# Supplementary material for: Suppression of Laccase 2 severely impairs cuticle tanning and pathogen resistance during the pupal metamorphosis of Anopheles sinensis (Diptera: Culicidae)
Source: Parasit Vectors. 2017 Apr 4;10:171. doi: 10.1186/s13071-017-2118-4 (PMC5381134; doi:10.1186/s13071-017-2118-4)
Supplement: Supplementary file 6 — Dataset 1. The identified Laccase 2A and the predicted Laccase 2B isoform in Anopheles sinensis. (DOCX 15 kb) [file 13071_2017_2118_MOESM6_ESM.docx]

Additional file 6

>Laccase 2A coding sequence (We cloned)

ATGGCCATAGACTGGCGAAATCGTGTGAACATAGCATCGCTCGGGATGCTGCTGCTGGTTGCCGTGGCCGCCGAAGCTGTCCGCGTCTCGCAGCACACATCCAGAAGATTCAAAGATGAATCGTTATCCCGTGACCAGAGTCCAGCCTCGTCCTGGTGGAGCTCACACCTGACCGAGCCACCAAGCAAGAACTTCTACCAGGCGACGCACGGTCTGCTGCAGACACACCCATCGGTGCCCGGTCTGAAGCCGGCCAGCCCATCGGCGCTGCCGCTCAGCTCGGCGGGCACTCGCAGCCCGACCGTCGGCGCACCACTGTCCTCGGGCAGCCTCAACAGTGGCTTTCCGTCCATTGCCAATCCCAACCCGCGGTCACCGTTTCGCCATCTGGACTTCAGCACGAGCGCGACGGCAGAGCTTCGCCGGAACCCGAGCCTCTCGGCGCCGGACGAGTGTGCCCGGGCGTGCCGCGAGGGAGAGCCGCCTAGGATTTGCTACTACCACTTCACCGTCGAGTACTACACCGTACTGGGAGCTGCCTGCCAGGTCTGCACACCGAACGCTACCAATACCGTCTGGAGTCACTGTCAGTGCGTTCTGGCCGACGGTGTCGAGCGCGGTATCCTCACGGTAAACCGTATGATCCCCGGACCGTCGATTCAGGTGTGCGAGAACGATCGCGTCGTGATCGACGTCGAGAACCACATGGAGGGCATGGAGCTGACGATCCACTGGCACGGCATCTGGCAGCGTGGCACGCAGTACTACGACGGTGTGCCGTTCGTCACGCAGTGTCCCATCCAGCAGGGCAACACCTTCCGTTATCAATGGACTGGCAACGCGGGTACGCACTTCTGGCACGCCCATACCGGTCTGCAGAAGCTGGACGGTCTGTACGGCAGCATCGTCGTGCGACAGCCACCGTCGCGCGACCCCAACTCGCACCTCTACGACTTCGATCTGACCACGCACATCATGCTGGTGAGCGACTGGCTGCACGAGGACGCGGCCGAACGCTACCCGGGCCGTCTGGCCGTCAACACCGGCCAGGATCCCGAGTCGCTGCTCATCAACGGCAAGGGCCAGTTCCGCGACCCGAACACCGGCTTCATGACCAACACGCCGCTGGAAATCTTCACCATCACGCCCGGTCGCCGCTACCGGTTCCGCATGATCAACGCGTTCGCGTCCGTCTGCCCGGCCCAGGTCACCATCGAGGGCCACGCCCTGACGGTGATTGCCACCGACGGCGAGCCGGTCCATCCGGTGCAGGTTAACACCATCATCTCGTTCTCCGGAGAACGTTACGATTTCGTCATCACCGCGGACCAACCGGTCGGTGCGTACTGGATCCAGCTGCGAGGACTCGGCGAATGCGGCATCAAGCGCGCCCAACAGTTGGGCATCCTTCGGTACGCCCGCGGACCGTACCAGCCCGCCTCCCCGCCACCCACGTACGACGTCGGTCTTCCGCAGGGTGTTGTCATGAACCCGCTCGATGCCCAGTGCAACGTTGAGCGGGACGATGCCATCTGCGTCAGCCAGCTGAAGAACGCCAAGGAGATCGACCGGGCGCTGCTGCAGGAGAAGCCGGACGTGAAGATTTTCCTGCCGTTCCGCTTCTATCTCTACCGCCCGGAGGAACTGTTTCAGCCGAACACGTACAACCGTTTCCTGGTCGCACCGACCGGAGACCATGTCATCTCGCTGATCGATGAGATCTCGTATCTGTCCGCACCGGCCCCACTCCTGTCGCAGTACGACGATATCAACCCGGAGCAGTTCTGCAACGGTGACAACCGGCCGGCCAACTGTGGTGCCAACTGCATGTGCACGCACAAGGTCGACATCCCACTGAACGCCATCGTGGAGGTCGTCCTGGTCGGCGAAGTCCAACAACCGAATCTGAGCCATCCGTTCCATCTGCACGGTTACGCGTACAACGTGATCGGTATCGGCCGCTCGCCGGACTCCAACGTGAAGAAGATTAACCTGAAGCACGCGCTCGACCTGGACCGACGCGGTCTGCTGCACCGACAGTACAATCTGCCCCCGCTCAAGGACACGATCGCCGTCCCCAACAATGGCTACGTGGTACTACGATTCCGTGCCGACAATCCAGGATTCTGGCTGTTCCACTGTCACTTCCTGTTCCACATCGTGATAGGAATGAACCTGATCCTGCAGGTCGGCACGATGGCCGATCTGCCGCCGGTGCCACCGAACTTCCCGACGTGCGGCGATCATCTGCCACCAATCAACTGA

> Laccase 2A protein sequence

MAIDWRNRVNIASLGMLLLVAVAAEAVRVSQHTSRRFKDESLSRDQSPASSWWSSHLTEP

PSKNFYQATHGLLQTHPSVPGLKPASPSALPLSSAGTRSPTVGAPLSSGSLNSGFPSIAN

PNPRSPFRHLDFSTSATAELRRNPSLSAPDECARACREGEPPRICYYHFTVEYYTVLGAA

CQVCTPNATNTVWSHCQCVLADGVERGILTVNRMIPGPSIQVCENDRVVIDVENHMEGME

LTIHWHGIWQRGTQYYDGVPFVTQCPIQQGNTFRYQWTGNAGTHFWHAHTGLQKLDGLYG

SIVVRQPPSRDPNSHLYDFDLTTHIMLVSDWLHEDAAERYPGRLAVNTGQDPESLLINGK

GQFRDPNTGFMTNTPLEIFTITPGRRYRFRMINAFASVCPAQVTIEGHALTVIATDGEPV

HPVQVNTIISFSGERYDFVITADQPVGAYWIQLRGLGECGIKRAQQLGILRYARGPYQPA

SPPPTYDVGLPQGVVMNPLDAQCNVERDDAICVSQLKNAKEIDRALLQEKPDVKIFLPFR

FYLYRPEELFQPNTYNRFLVAPTGDHVISLIDEISYLSAPAPLLSQYDDINPEQFCNGDN

RPANCGANCMCTHKVDIPLNAIVEVVLVDEVQQPNLSHPFHLHGYAYNVIGIGRSPDSNV

KKINLKHALDLDRRGLLHRQYNLPPLKDTIAVPNNGYVVLRFRADNPGFWLFHCHFLFHI

VIGMNLILQVGTMADLPPVPPNFPTCGDHLPPIN

>Predicted Laccase 2B coding sequence

ATGGCCATAGACTGGCGAAATCGTGTGAACATAGCATCGCTCGGGATGCTGCTGCTGGTT

GCCGTGGCCGCCGAAGCTGTCCGCGTCTCGCAGCACACATCCAGAAGATTCAAAGATGAA

TCGTTATCCCGTGACCAGAGTCCAGCCTCGTCCTGGTGGAGCTCACACCTGACCGAGCCA

CCAAGCAAGAACTTCTACCAGGCGACGCACGGTCTGCTGCAGACACACCCATCGGTGCCC

GGTCTGAAGCCGGCCAGCCCATCGGCGCTGCCGCTCAGCTCGGCGGGCACTCGCAGCCCG

ACCGTCGGCGCACCACTGTCCTCGGGCAGCCTCAACAGTGGCTTTCCGTCCATTGCCAAT

CCCAACCCGCGGTCACCGTTTCGCCATCTGGACTTCAGCACGAGCGCGACGGCAGAGCTT

CGCCGGAACCCGAGCCTCTCGGCGCCGGACGAGTGTGCCCGGGCGTGCCGCGAGGGAGAG

CCGCCTAGGATTTGCTACTACCACTTCACCGTCGAGTACTACACCGTACTGGGAGCTGCC

TGCCAGGTCTGCACACCGAACGCTACCAATACCGTCTGGAGTCACTGTCAGTGCGTTCTG

GCCGACGGTGTCGAGCGCGGTATCCTCACGGTAAACCGTATGATCCCCGGACCGTCGATT

CAGGTGTGCGAGAACGATCGCGTCGTGATCGACGTCGAGAACCACATGGAGGGCATGGAG

CTGACGATCCACTGGCACGGCATCTGGCAGCGTGGCACGCAGTACTACGACGGTGTGCCG

TTCGTCACGCAGTGTCCCATCCAGCAGGGCAACACCTTCCGTTATCAATGGACTGGCAAC

GCGGGTACGCACTTCTGGCACGCCCATACCGGTCTGCAGAAGCTGGACGGTCTGTACGGC

AGCATCGTCGTGCGACAGCCACCGTCGCGCGACCCCAACTCGCACCTCTACGACTTCGAT

CTGACCACGCACATCATGCTGGTGAGCGACTGGCTGCACGAGGACGCGGCCGAACGCTAC

CCGGGCCGTCTGGCCGTCAACACCGGCCAGGATCCCGAGTCGCTGCTCATCAACGGCAAG

GGCCAGTTCCGCGACCCGAACACCGGCTTCATGACCAACACGCCGCTGGAAATCTTCACC ATCACGCCCGGTCGCCGCTACCGGTTCCGCATGATCAACGCGTTCGCGTCCGTCTGCCCG

GCCCAGGTCACCATCGAGGGCCACGCCCTGACGGTGATTGCCACCGACGGCGAGCCGGTC

CATCCGGTGCAGGTTAACACCATCATCTCGTTCTCCGGAGAACGTTACGATTTCGTCATC

ACCGCGGACCAACCGGTCGGTGCGTACTGGATTCAGCTGCGAGGACTCGGCGAATGCGGC

ATCAAGCGCGCCCAACAGTTGGGCATCCTTCGGTACGCCCGCGGACCGTACCAGCCCGCC

TCCCCGCCACCCACGTACGACGTCGGTCTTCCGCAGGGTGTTGTTCTAAACCCGCTCGAT

GCCGTCTGCAACGTACCCCGGCCGGATGCGATCTGCGTGAGCAACCTGCGGAACGCCAAG

AAGGCCGACAAGGCCGTCCTCTCCGAGCGACCCGACGTGAAGATTTTCCTACCGTTCCGG

TTCTACTTCTACCGCGTCGAGGAGCTGTTCACGCCGAACACGTACAACAAATTTTTGGTT

GCACCGGGAGGAGACCACCTGATTTCGCTGATCGACGAAATCTCCTACGTATCGCCACCG

TCTCCGATGCTGTCGCAGATCAACGACATCCCACCGGAGCAGTTCTGCAACGGTGACAAC

CGCCCGCCAGACTGCGGCCCGAACTGCATGTGCACGCACAAGGTCGACATTCCGCTGAAC

GCCATCGTGGAGGTCGTCCTGGTCGACGAAGTACAACAAGACAATCTGAGCCATCCGTTC

CATCTACACGGACACGCGTTCCACGTGATCGGTATGGGCCGCTCGCCGGACAGCACCGTG

AAGAAGATCAACCTGCGCCACACGCTCGACCTGGACCGACGGGGTCTGCTGAACCGACAG

TTTAACCTTCCCCCTCTTAAGGACACGATCGCCGTCCCCAACAATGGCTACGTGGTACTA

CGATTCCGGGCAGATAATCCCGGATATTGGTTGTTCCACTGCCACTTCCAGTTCCACATA

GTGATCGGCATGAATCTGGTGGTGCACATCGGCACCCACGCAGACCTTCCACCGGTGCCA

CCGAACTTCCCCCGCTGCGGAAACCATATACCTCCGATTAAGTTTAACTAG

> Predicted Laccase 2B protein sequence

MAIDWRNRVNIASLGMLLLVAVAAEAVRVSQHTSRRFKDESLSRDQSPASSWWSSHLTEP

PSKNFYQATHGLLQTHPSVPGLKPASPSALPLSSAGTRSPTVGAPLSSGSLNSGFPSIAN

PNPRSPFRHLDFSTSATAELRRNPSLSAPDECARACREGEPPRICYYHFTVEYYTVLGAA

CQVCTPNATNTVWSHCQCVLADGVERGILTVNRMIPGPSIQVCENDRVVIDVENHMEGME

LTIHWHGIWQRGTQYYDGVPFVTQCPIQQGNTFRYQWTGNAGTHFWHAHTGLQKLDGLYG

SIVVRQPPSRDPNSHLYDFDLTTHIMLVSDWLHEDAAERYPGRLAVNTGQDPESLLINGK

GQFRDPNTGFMTNTPLEIFTITPGRRYRFRMINAFASVCPAQVTIEGHALTVIATDGEPV

HPVQVNTIISFSGERYDFVITADQPVGAYWIQLRGLGECGIKRAQQLGILRYARGPYQPA

SPPPTYDVGLPQGVVLNPLDAVCNVPRPDAICVSNLRNAKKADKAVLSERPDVKIFLPFR

FYFYRVEELFTPNTYNKFLVAPGGDHLISLIDEISYVSPPSPMLSQINDIPPEQFCNGDN

RPPDCGPNCMCTHKVDIPLNAIVEVVLVDEVQQDNLSHPFHLHGHAFHVIGMGRSPDSTV

KKINLRHTLDLDRRGLLNRQFNLPPLKDTIAVPNNGYVVLRFRADNPGYWLFHCHFQFHI

VIGMNLVVHIGTHADLPPVPPNFPRCGNHIPPIKFN
