# Supplementary figures and images for: Suppression of Laccase 2 severely impairs cuticle tanning and pathogen resistance during the pupal metamorphosis of Anopheles sinensis (Diptera: Culicidae)
Source: Parasit Vectors. 2017 Apr 4;10:171. doi: 10.1186/s13071-017-2118-4 (PMC5381134; doi:10.1186/s13071-017-2118-4)

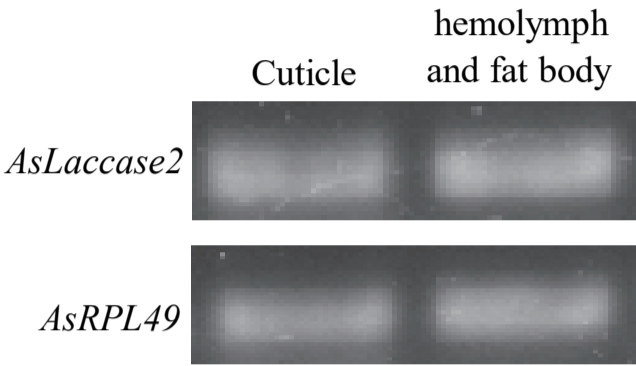

Supplement: Supplementary file 7 — Gene expression patterns of AsLac2 in pupal cuticle, fat body, and hemolymph. AsRPL49 was used as the internal control. (PDF 72 kb) [file 13071_2017_2118_MOESM7_ESM.pdf]
